# Predicting neural responses to natural sound in the auditory brainstem

**DOI:** 10.1186/1471-2202-15-S1-O6

**Published:** 2014-07-21

**Authors:** Dominika Lyzwa, Michael Herrmann

**Affiliations:** 1 Dept. of Nonlinear Dynamics, Max Planck Inst. for Dynamics and Self-Organization, Göttingen, 37077, Germany; 2 Institute of Perception, Action and Behaviour, University of Edinburgh, Edinburgh, EH 8 9AB, UK

## 

The inferior colliculus is the main processing station in the auditory midbrain and integrates projections from nearly all ascending brainstem nuclei. Apart from being a converging station, the central nucleus of the inferior colliculus (ICC) is essential for extracting time-varying spectrotemporal information [1] and therefore might be important for processing complex sounds such as speech and vocalizations. The ICC has been the target for a human auditory prosthesis [2], which might benefit from model predictions of the neural response in the ICC to incoming sound.

Natural sounds such as speech and vocalizations, which display a wide spectrum of acoustic properties, such as harmonics, correlations, amplitude and frequency modulations and are very well suited to study the auditory system.

This study is based on several sets of multi-unit activity recorded simultaneously from 32 sites in the contralateral ICC of guinea pigs while acoustically presenting a diverse set of conspecific vocalizations to the right ear. Recordings were taken either along the tonotopic gradient using double-shank electrodes or within iso-frequency lamina using double-tetrode electrodes.

We investigated predictive power of several models of temporal responses in the ICC to vocalizations and artificial sound. The tested models include 1) a modified version of the physiologically detailed Meddis Model [4], which was altered in order to match spiking threshold in the guinea pig ICC and to include adaptation effects and output the trial-averaged spiking responses, the peri-stimulus time histograms (PSTH).

2) a generalized linear model and 3) a filtering model with a bandpass filter of 1/3 octaves around the best frequency, with subsequent normalization and rectification for each unit, followed by spatial filtering for nearby units. Predictive power was evaluated by means of the correlation value of the envelope of the PSTHs from the predicted and the experimentally obtained responses.

We find that our relatively simple, filtering approach yields surprisingly good overlap of predicted and measured responses for some multi-units, but has poor predictive power for other units. The models (1-2) yield overall better overlap for the whole set of vocalizations but do not perform optimally in predicting the temporal course of the response.

Our findings indicate distributions of optimal predictive power in the inferior colliculus over a large best frequency range across and within isofrequency laminae.
